# Induction of Apoptosis in *Toxoplasma gondii* Infected Hela Cells by Cisplatin and Sodium Azide and Isolation of Apoptotic Bodies and Potential Use for Vaccination against *Toxoplasma gondii*

**Published:** 2018

**Authors:** Kourosh CHERAGHIPOUR, Laleh SHARIATI, Hossein KHANAHMAD, Mazdak GANJALIKHANI-HAKEMI, Abbas MORIDNIA, Mina MIRIAN, Nader PESTEHCHIAN

**Affiliations:** 1. Dept. of Parasitology and Mycology, School of Medicine, Isfahan University of Medical Sciences, Isfahan, Iran; 2. Razi Herbal Medicines Research Center, Lorestan University of Medical Sciences, Khorramabad, Iran; 3. Isfahan Cardiovascular Research Center, Cardiovascular Research Institute, Isfahan University of Medical Sciences, Isfahan, Iran; 4. Pediatric Inherited Diseases Research Center, Research Institute for Primordial Prevention of Non-Communicable Diseases, Isfahan University of Medical Sciences, Isfahan, Iran; 5. Dept. of Genetics, School of Medicine, Isfahan University of Medical Sciences, Isfahan, Iran; 6. Dept. of Immunology, School of Medicine, Isfahan University of Medical Sciences, Isfahan, Iran; 7. Dept. of Immunology, School of Medicine, Dezful University of Medical Sciences, Dezful, Iran; 8. Dept. of Pharmaceutical Biotechnology, School of Pharmacy and Pharmaceutical Sciences, Isfahan University of Medical Sciences, Isfahan, Iran; 9. Infectious Diseases and Tropical Medicine Research Center, Isfahan University of Medical Sciences, Isfahan, Iran

**Keywords:** Apoptosis, Apoptotic blebs, *Toxoplasma gondii*, Toxoplasmosis, Cisplatin, NaN3

## Abstract

**Background:**

*Toxoplasma gondii* can infect a wide range of mammalians, especially humans. It controls several intracellular signals for the inhibition of apoptosis. This study aimed to investigate the apoptogenic effect of cisplatin and sodium azide on *T. gondii* infected HeLa cells and isolate apoptotic bodies (blebs) as a potent stimulator of the immune system.

**Methods:**

*T*he cytotoxic properties of cisplatin and sodium azide (NaN3) on HeLa cells were evaluated by MTT assay. Moreover, the apoptogenic activity of cisplatin and NaN3 was studied using flow cytometry (Annexin V/PI double staining) and scanning electron microscopy (SEM). Finally, apoptotic bodies were separated by centrifugation.

**Results:**

MTT assay data showed that the survival rate of cells treated with different concentration of NaN3 was significantly reduced, compared to negative control groups. Concerning cisplatin, only concentration of 20 μM had not a significant impact on the cell viability; however, the other concentration of cisplatin significantly reduced cell viability, compared to negative control groups. The level of early apoptosis in uninfected HeLa cells was higher compared to infected HeLa cells treated with cisplatin and NaN3. Finally, apoptotic bodies were separated from T. gondii infected HeLa cells treated with cisplatin.

**Conclusion:**

Apoptosis was induced in both uninfected and infected HeLa cells with T. gondii and apoptotic bodies were isolated from infected cells. Therefore, further studies on apoptotic bodies are required in order to find a proper candidate for vaccine preparation against *T. gondii* infections.

## Introduction

*Toxoplasma gondii* is one of the most frequent protozoan parasites which can infect a wide range of mammalians, especially humans. Humans are generally infected by *T. gondii* through eating raw meat and undercooked containing tissue cysts or by ingestion of oocysts. Toxoplasmosis is presumably the most regular food-related disease in the most areas of the world (1). Organ transplantation and congenital transmission are also other courses of the infection (2).

*T. gondii* infection can cause stillbirth, abortion and neonatal loss in pregnant women (3). During pregnancy, if an immunocompetent mother is infected for the first time, she may abort her child. *Toxoplasma* could cause a disseminated disease in patients that have impaired T-cell immunity (4). Available medications for prevention and treatment of toxoplasmosis have shown limited efficacy or substantial side effects (5).

*T. gondii* has affected almost one billion individuals worldwide and it is the most common parasitic disease, but no effective therapy has been found in the early stage of this infection (6). Apoptosis is a controlled and programmed cell death, which leads to the elimination of unhealthy cells and retention of healthy environment for cells in the body (7). Intracellular pathogens have evolved various strategies to evade the host immune system. *T. gondii* and its derivatives are able to increase and decrease the gene expression level of IL-10 in a murine model. The question remains to be examined in further study about which molecules are involved in this process. Apoptosis is one of the main mechanisms for eliminating infected host cells. *T. gondii*, an intracellular parasite, is able to cause infection in almost all warm-blooded animals (8). *T. gondii* may prevent the release of cytochrome C from infected cells and hence, suppress the process of internal apoptosis (9). In addition, *T. gondii* can cause the modulation of apoptosis in infected host cells (10).

*T. gondii* may control apoptosis. It may interfere with signal pathways that regulate cell survival, including caspase 3 activation, PARP-1 or cytochrome C release from the mitochondria. It may also stimulate anti-apoptotic gene expression or prevent expression of pro-apoptotic genes, finally leading to inhibition of DNA fragmentation (11). Cisplatin as a powerful platinum-based antineo-plastic agent seems to create inter- and intra-strand DNA adducts which activate signal pathways culminating in apoptosis. This compound has additionally been shown to induce apoptosis through caspase-3 activation and X-linked inhibitor-of-apoptosis protein XIAP expression (12, 13). On the other hand, sodium azide, an inhibitor of complex IV, may induce apoptosis in primary cortical neuronal cells. This is caspase-3-dependent and promotes the release of cytochrome C (14). Caspase-3 is normally placed in the cytoplasm as a precursor. After its proteolytic cleavage by the cytochrome C, it converts caspase-9 and APAF1 complex to their active forms (15). Cisplatin can cause apoptosis and death in HeLa cells. Through this mechanism, it will be able to up-regulate Bax in HeLa cells (16). Apoptosis analysis using electron microscopy can be the best option for studying this mechanism and to distinguish it from necrosis (17).

We aimed to compare the apoptogenic effect of cisplatin and Sodium azide on *T. gondii* infected HeLa cells and also to isolate apoptotic bodies (blebs) as a potent stimulator of the immune system.

## Materials and Methods

This study was conducted in Isfahan University of Medical Sciences in 2016. Approval of Ethics Committee of all patients participating in the study were obtained IR.Iums.REC.394228.

### The HeLa cell line

The HeLa cell line was provided by Pasteur Institute of Iran. The cells were cultured at 37 °C in Roswell Park Memorial Institute (RPMI1640) (Sigma-Aldrich, USA) containing 100 U/ml of penicillin and 100 μg/ml streptomycin, and supplemented with 10% fetal calf serum (Sigma-Aldrich, USA).

### Preparation of T. gondii tachyzoites

Virulent RH strain of *T. gondii* was provided from Pasteur Institute of Iran. For parasite propagation, virulent RH strain of *T. gondii* tachyzoites was injected into the BALB/c mice peritoneal *cavity*. Three days after inoculation, peritoneal fluids were extracted and collected in cold PBS. Centrifugation was performed in order to isolate the tachyzoites.

### Preparation of cisplatin and NaN3

At first according to MW of cisplatin (300.0 g/mol) and NaN3 (65.00987 g/mol) 300 mg of cisplatin and 60 mg of NaN3 were solved in 10ml of DMEM in separate tubes to prepare 100 mM master drug solution then for preparation of each concentration the calculated volume of this master solution was added to culture medium to achieve related final concentration. Master solution of Cisplatin and NaN3 Should be kept in a tightly closed container, protected from light, and stored at a temperature between 2 and 8 °C.

### In vitro cultivation of T. gondii strains

*Cultivation* of *T. gondii* isolated from peritoneal fluid of mice in *HeLa cells* was performed in a 1 to 1 multiplicity of infection *ratio (18).*

### Cell viability assay

The different concentrations of cisplatin and NaN3 on HeLa cells was examined using MTT assay, in order to select the best concentration with most apoptogenic activity. The diphenyl tetrazolium bromide (MTT) assay acts based on the conversion of MTT to formazan crystals in living cells which determines the mitochondrial enzyme activity. In the MTT assay, HeLa cells were categorized into three groups: 1. Test group composed of the cells treated with different concentration of NaN3 (1, 2.5, 5, 10, 15 and 20 μM) in one experiment and different concentration of cisplatin (20, 25, 50, 100 and 200 and 250 μM) in another experiment. 2. The negative control group, including untreated cells. 3. The blank group containing medium without cells. After 24 h of plating 5×10^5^ HeLa cells on each well of 12-well plates, the supernatant was replaced with fresh medium containing cisplatin or NaN3 in different concentration and 10% fetal bovine serum (FBS) (Bovogen, Australia). After 24 h, MTT assay was performed according to the in vitro Toxicology Assay Kit (Sigma-Aldrich, USA) instruction. The optical density was recorded at 570 nm filter by an ELISA microplate reader (Hyperion, USA). Percentage of cell survival was calculated using the following equation:

$$\%\;\text{Cell}\;\text{survival}=\frac{\text{Mean}\;\text{absorbance}\;\text{of}\;\text{treated}\;\text{cells}-\;\text{Mean}\;\text{absorbance}\;\text{of}\;\text{blank}}{\text{Mean}\;\text{absorbance}\;\text{of}\;\text{untreated}\;\text{cells}-\;\text{Mean}\;\text{absorbance}\;\text{of}\;\text{blank}}\times 100$$

### Annexin V-FITC Assay

In this step, HeLa cells were grouped into three sets. First set was treated with 25μM cisplatin. The second set was infected with a RH strain of T. *gondii* tachyzoites for 1 h, followed by treatment with 25 μM cisplatin (Toxoplasma+cisplatin 25μM) and the third group was treated with 25μM cisplatin, followed by a 1-hour infection with RH strain of *T. gondii* tachyzoites. The same study design was applied to HeLa cells treatment with NaN3. Plates were incubated at 37°C, 5% CO2, and 95% humidity. After 12 h, cells in each well were collected by trypsinized and centrifuged. The pellet of each well was resuspended in a final volume of 100 μL in 1x incubation buffer and transferred to the distinct flow cytometric tube. Then, Annexin V-FITC and propidium Iodide (PI) were added to each tube based on manufacturer’s instruction (Roche, Germany). Annexin-V was used to conjunct to the phosphatidylserine of apoptotic cells while necrotic cells were stained with Propidium iodide (PI). The HeLa cells were stained with Annexin V and PI as a control tube and were incubated at 25 °C for 15 min in darkness. After incubation, 500 μL of incubation buffer was added to each tube. Eventually, the HeLa cells were analyzed with a FACS Calibur flow cytometer (Becton Dickinson, USA).

### Exterior ultra-structural effects of cisplatin on HeLa cells

Cisplatin was used to induce apoptosis in HeLa cells. *T. gondii* infected and uninfected HeLa cells were exposed to (25μM) cisplatin for 12 h. Cells were trypsinized and centrifuged at 300 g for 10 min. The pellets were fixed in 2.5% glutaraldehyde for 4 h at room temperature. The fixed cells were washed three times with PBS; 10 min for each time. Then, dehydration process was performed in alcohol ascending grades (50%, 70%, 80%, 90% and 100% V/V) for 10 min. Then, they brought to the critical point of drying by the critical point dryer (S4160, Hitachi, Japan) for thirty minutes. The cells were fixed on a metal scanning electron microscopy (SEM) stub and sputter coated in gold using SEM coating unit (E5100 Polaron, UK). The coated specimen was investigated using scanning electron microscopy (JOEL 64000, Japan) at an acceleration voltage of 15–25 KV.

### Bleb isolation from cell culture

Separation of the blebs was performed using different centrifugation steps. Initially, centrifugation at 300 g was done in order to remove the cells, then, centrifugation of the supernatant at 16500 g for 20 min was performed to collect blebs (19).

### Statistical analysis

Data presented in this work are expressed as mean ± SE and experiments were repeated three times. Normality and homogeneity of variance assumptions were checked. Information was analyzed by Social Science Software (SPSS for Windows, ver. 20.0, Chicago, IL, USA). Statistical significance among the experiments was evaluated by one way ANOVA followed by Duncan’s multiple range tests. The significance level was set at *P*<0.05.

## Results

### Cell viability assay

**Fig. 1:**
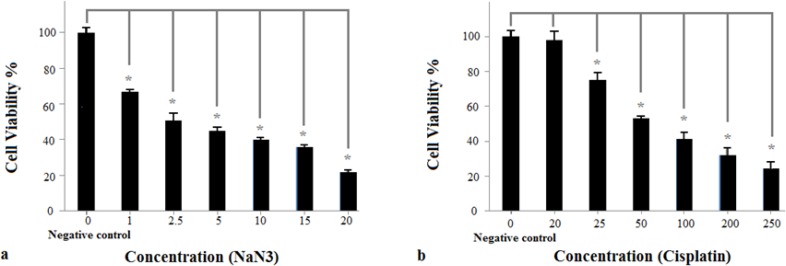
Evaluation of cell viability using the MTT assay **A)** Effects of various concentrations of NaN3 on the viability of the HeLa cells after 24 h. The viability of the cells treated with various concentrations of NaN3 had significant difference compared to untreated negative control cells (^*^P<0.05). Data are presented as mean±SEM of three identical repeats of each experiment **B)** Effects of various concentrations of cisplatin on the viability of the HeLa cells after 24 h. The viability of the cells treated with various concentrations of cisplatin had significant difference compared to untreated negative control cell except the group that treated with 20μM cisplatin (^*^P<0.05).

Cell survival rate in cells treated with all concentration of NaN3 was significantly reduced, compared to corresponding values in negative control group (*P*<0.05) (Fig. 1-a). Regarding cisplatin, cell survival rate in cells treated with all different concentration was significantly reduced compared to negative control group except cell survival rate related to concentration of 20μM (*P*<0.05) (Fig. 1-b, Table 1).

**Table 1:** Effects of NaN3 and cisplatin on the viability of HeLa cells

***Treatment (NaN3)***	***Negative control***	***1***	***2.5***	***5***	***10***	***15***	***20***
Cells viability (mean±SEM)	100±3.01	67±1.02	51±4.03	45±2.30	40±1.16	36±1.17	22±1.28
Treatment (Cisplatin)	Negative control	20	25	50	100	200	250
Cells viability (mean±SEM)	100±3.43	98±5.10	75±4.21	53±1.50	41±3.98	32±4.01	24±4.01

### Flow cytometry analysis of apoptosis in HeLa cells after different treatments

**Fig. 2:**
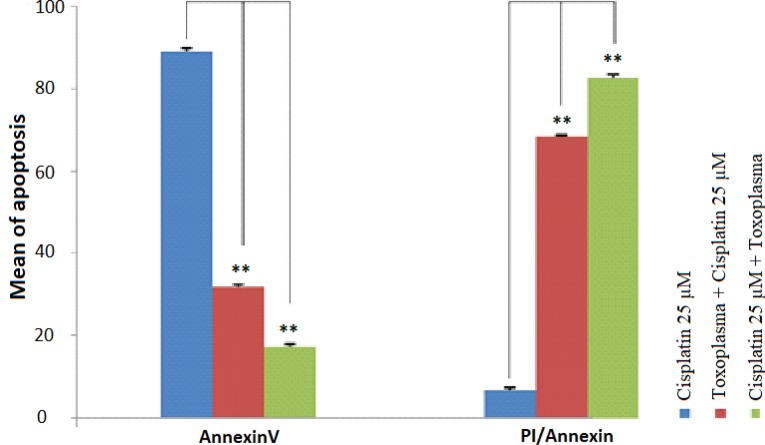
Comparison of the early and late apoptosis in HeLa cells treated with cisplatin, *Toxoplasma*+Cisplatin 25μM and Cisplatin 25μM+*Toxoplasma* Cells stained with Annexin V represent an early stage of apoptosis. The most cells in the early apoptotic stage were seen in cisplatin-treated cells. However, cells treated with cisplatin 25μM +*Toxoplasma* showed the most level of the late stage of apoptosis. The early apoptosis significantly decreased in *Toxoplasma* +Cisplatin 25μM and Cisplatin 25μM +*Toxoplasma* groups vs. cisplatin group, ^**^P<0.001. The late apoptosis significantly increase in *Toxoplasma* +Cisplatin 25μM and Cisplatin 25μM +*Toxoplasma* groups vs. cisplatin group, ^**^P<0.001

**Fig. 3:**
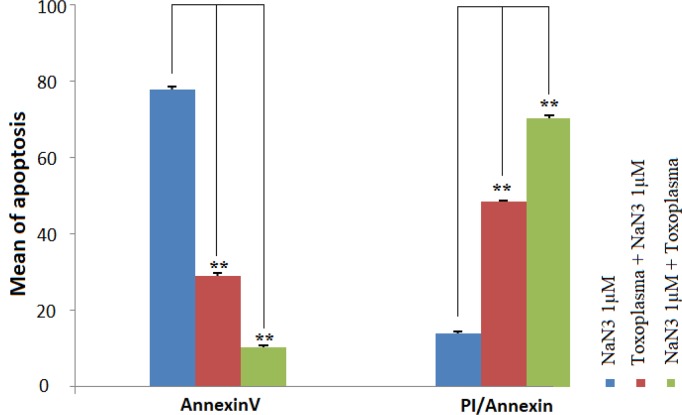
Comparison of the early and late apoptosis in HeLa cells treated with NaN3, Toxoplasma+NaN3 1μM, and NaN3 1μM + Toxoplasma Cells stained with Annexin V represent the early stage of apoptosis. The most cells in the early stage of apoptosis were seen in NaN3 treated cells. However, cells treated with NaN3 +*Toxoplasma* showed the most level of the late stage of apoptosis. Early apoptosis significantly decreased in Toxoplasma+NaN3 and NaN3 +*Toxoplasma* groups vs. NaN3 group, ^**^P<0.001. The late apoptosis significantly increase in Toxoplasma+NaN3 and NaN3 +*Toxoplasma* groups vs. NaN3 group, ^**^P<0.001

**Fig. 4:**
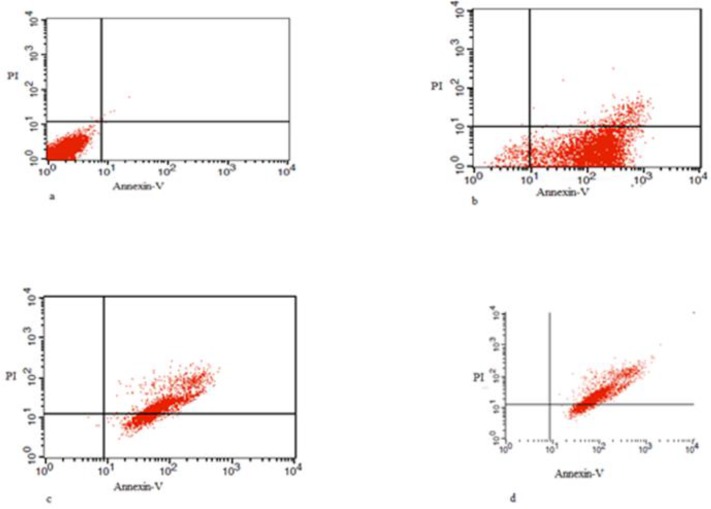
Dot plot diagrams of Cisplatin **(a)** Dot plot diagrams showed in untreated the HeLa cells as control group; **(b)** The highest level of early apoptosis 88.9% and late apoptosis 6.6% in the HeLa cells induced with 25μM Cisplatin after 12 h; **(c)** Dot plot diagrams showed the level of early late apoptosis equal to 68.3% in the HeLa cells treated with the *Toxoplasma* + Cisplatin 25μM after 12 h; **(d)** The late apoptosis equal to 85.7% in the HeLa cells treated with 25 μM of Cisplatin + *Toxoplasma* after 12 h

Annexin-V was utilized to detect apoptotic bodies in HeLa cells. We got less than 1% cell death in the untreated control cells. The highest percentage of early apoptosis was observed in the concentration of 25 μM of cisplatin (88.9%). Also, the lowest level of necrosis was observed in HeLa cells treated with only 25μM cisplatin (6.6%). When cells first infected by *T. gondii* and then treated with cisplatin, the early apoptosis rate was significantly decreased (*P*<0.001). When the HeLa cells were first treated with cisplatin and then *T. gondii* was added, the most of the cells undergoing the late apoptosis process (82.7%). Moreover, dot plot diagrams of cisplatin showed that the highest percent of apoptosis was in non-infected HeLa cells (88/9%) (Figs. 2–4). Cisplatin has higher apoptosis rate (88.9%) compared to NaN3, hereafter cisplatin has been used for induction of apoptotic bodies.

### Cisplatin affected the ultra-structure of HeLa cells

**Fig. 5:**
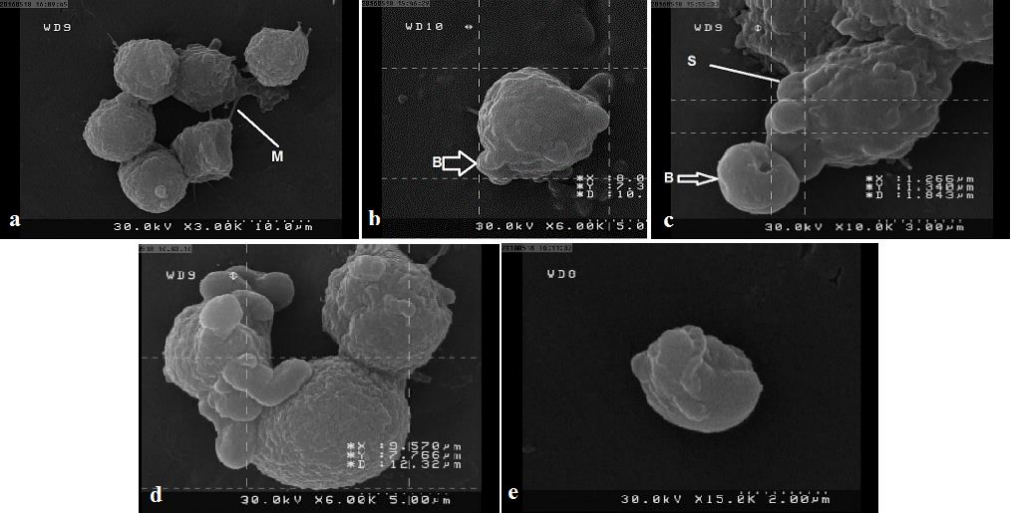
SEM micrographs of surface ultra-structural characteristics of HeLa cells treated with cisplatin for 12 h (a) Control HeLa cells surface showed the restoration of a typical morphological feature of cancer cell such as numerous microvilli with several membrane connections indicated by white arrow (M). (b) Treated HeLa cells with *Toxoplasma* + Cisplatin (12 h, IC50: 41μgr/ml). (c and d) Treated HeLa cells with cisplatin without *T. gondii* showed distinct morphological changes corresponding to typical apoptosis, including cell membrane blebbing (B) and cytoplasmic extrusions (S) (b, c, d). (e) Blebs were isolated from apoptotic HeLa cells

The surface ultrastructure of HeLa cells treated with cisplatin was evaluated with SEM. Untreated HeLa cells have shown a restoration of the typical morphological features of cervical cancer cells which include numerous microvilli on the surface with membrane connections in Fig. 5(a). The effect of cisplatin on *T. gondii* (RH strain) infected HeLa cells and uninfected HeLa cells are shown in Fig. 5(b, c, d). The presence of blebs in cells was an indicator of cell death via apoptosis (Fig. 5). Blebs were isolated from apoptotic HeLa cells. Although, the occasional existence of rounded cells and blebbing on the cell surface are characteristics of Hela cells in culture this phenomenon is not common. Here, there is a bleb production process both in *T. gondii* infected HeLa cells and in uninfected HeLa cells.

## Discussion

Almost 25% of the human population is at risk of toxoplasmosis infection (20). This infection, not only may cause abortion in women during their first pregnancy, it can also cause encephalitis and other problems related to the infection in immunocompromised individuals (21). *Toxoplasma* is an obligate parasite in all vertebrates that can infect all nucleated cells (22). *Toxoplasma* controls several intracellular signals for the inhibition of apoptosis. This process will lead to the parasite survival in cells and interfere with the effective immune response against parasites (23). In fact, *T. gondii* has several strategies for inhibiting the initiation of the apoptotic cascade (9). We have shown that the apoptosis rate was higher in uninfected compared to infected HeLa cells. In parasites, the mechanism of inhibition of apoptosis is not clear. Upregulation of pro-apoptotic proteins and inhibition of NFκB, AP1, and their target genes may lead to induction of apoptosis in HeLa cells (24). Upregulation of the expression of anti-apoptotic Bcl-2 family members and inhibitors of apoptosis (IAP) can occur via infection with *Toxoplasma* (25). Additionally, it controls several cellular pathways to establish an anti-apoptotic environment (23). Apoptosis will be inhibited in *T. gondii* infection through inactivation of caspase and NF-κB activation in cells (26). Another study also confirms our data in regards to decreasing in apoptosis rate in infected compared to uninfected cells (27).

Based on our results, NaN3 and cisplatin showed the ability of apoptosis induction in HeLa cells. Cisplatin induces apoptosis using cell cycle arrest in G1 phase (28). The cytotoxic assay showed NaN3 leads to less cell viability compared to cisplatin. Cisplatin led to more cell viability compared to other compounds (24). Early and late apoptotic cells were measured by flow cytometric analysis after double staining with FITC-conjugated Annexin V and PI (29). Our results showed that treated HeLa cells by cisplatin and NaN3 could induce apoptosis in both infected and uninfected cells. We demonstrated that concomitant infection with *T. gondii* reduced cisplatin and NaN3 induced apoptosis. The cisplatin and NaN3 induced apoptosis in treated population was 88.9% and 77.7%, respectively. On the other hand, cisplatin and NaN3 induced apoptosis in *T. gondii* infected HeLa cells was as much as 31.7% and 28.9%, respectively. *T. gondii* may control apoptosis in induced astrocytes by cisplatin (27). When the cells treated with both actinomycin-D (AD) and *T. gondii*, early apoptotic cells are fewer than the cells treated only by actinomycin-D (AD)(26). Conjugated linoleic acid could lead to apoptosis in RH strain *T. gondii* infected HeLa cells and has an anti-toxoplasmacidal function on tachyzoites (18). In the current work, morphological studies show some evidence in regards to cisplatin treatment which leads to an apoptotic phenomenon. SEM was applied to take precise information about changes in the cell surface such as erosion surface microvilli, production of apoptotic bodies and membrane connections in the infected HeLa cells after treatment with cisplatin. Signs of apoptosis in Hela cell were in accordance with another experience (30).

In this work and for the first time SEM technique has been used to assess the infected HeLa cells by *T. gondii. Toxoplasma* sustain itself in cells using different survival mechanisms such as inhibition of apoptosis (11). Commentary of SEM electro-micrograph showed distinct morphological changes similar to a typical cellular surface morphology of apoptosis including cell membrane blebbing, microvilli disappearance or reduction and separation of apoptotic bodies (30).

Induction of apoptosis in infected cells leads to ultra-morphological changes. Apoptotic bodies are direct byproducts of apoptosis and cross-priming through them has been demonstrated previously, to be relevant in antiviral and tumor immunity. The lytic function of CD 8 T cells, performed though MHC I and CD1 and also by cross-presentation of intra-cellular pathogen antigens has been facilitated by apoptosis as a survival mechanism to enhance anti-bacterial immunity in the cells (31). The immunization with apoptotic cells was examined in tumor models in order to induce a cytotoxic immune response (32). In another study, immunization with apoptotic phagocytes containing heat-killed Histoplasma activated CD8+ T cells was examined (33). Utilization of the apoptotic bodies derived from *T. gondii* infected HeLa cells can be used as a vaccine to stimulate the cross-priming mechanism. Various products that contribute to increase apoptosis and improve immune responses might be able to increase our resistance against invading parasites.

## Conclusion

Apoptosis induces effects of cisplatin and NaN3 on infected HeLa cells. Finally, apoptotic bodies were isolated from *T. gondii* infected HeLa cells. Regarding the role of apoptotic body in cross-priming, there is a potential use for vaccine preparation against *T. gondii.* Further studies required in order to examine the vaccination of mouse model by apoptotic bodies a crucial step, before performing initial *clinical trials* in humans.
